# Evaluation of Phenotypic and Genotypic Susceptibility Testing Methods for Newer β-lactam/β-lactamase Inhibitor Combinations in Multidrug Resistant *Pseudomonas aeruginosa*

**DOI:** 10.1093/infdis/jiaf585

**Published:** 2025-11-17

**Authors:** Clayton W Hall, Nicholas Waglechner, Erin Choi, Patryk Aftanas, Kevin Katz, Christie Vermeiren, Finlay Maguire, Robert Kozak, Xena X Li

**Affiliations:** Medical Microbiology, Department of Pathology and Molecular Medicine, McMaster University, Hamilton, ON, Canada; Shared Hospital Laboratory, Toronto, ON, Canada; Shared Hospital Laboratory, Toronto, ON, Canada; Shared Hospital Laboratory, Toronto, ON, Canada; Shared Hospital Laboratory, Toronto, ON, Canada; Laboratory Medicine and Molecular Diagnostics, Sunnybrook Health Sciences Centre, Toronto, ON, Canada; Department of Laboratory Medicine and Pathobiology, University of Toronto, Toronto, ON, Canada; Shared Hospital Laboratory, Toronto, ON, Canada; Department of Laboratory Medicine and Pathobiology, University of Toronto, Toronto, ON, Canada; Shared Hospital Laboratory, Toronto, ON, Canada; Department of Community Health and Epidemiology, Faculty of Medicine, Dalhousie University, Halifax, NS, Canada; Faculty of Computer Science, Dalhousie University, Halifax, NS, Canada; Shared Hospital Laboratory, Toronto, ON, Canada; Laboratory Medicine and Molecular Diagnostics, Sunnybrook Health Sciences Centre, Toronto, ON, Canada; Department of Laboratory Medicine and Pathobiology, University of Toronto, Toronto, ON, Canada; Shared Hospital Laboratory, Toronto, ON, Canada; Laboratory Medicine and Molecular Diagnostics, Sunnybrook Health Sciences Centre, Toronto, ON, Canada

**Keywords:** *Pseudomonas aeruginosa*, antibiotic resistance, ceftazidime-avibactam, ceftolozane-tazobactam, imipenem-relebactam

## Abstract

**Background:**

Ceftazidime-avibactam (CZA), ceftolozane-tazobactam (CT), and imipenem-relebactam (IMR) are newer β-lactam/β-lactamase inhibitor (BL/BLI) combinations used for treatment of multidrug resistant (MDR) *Pseudomonas aeruginosa*, although resistance has already emerged. Optimal use of these agents relies on timely, accurate susceptibility testing results and understanding of local resistance mechanisms.

**Methods:**

183 MDR *P. aeruginosa* clinical isolates were used to evaluate the performance of commercial Sensititre and Phoenix panels for CZA, CT, and IMR susceptibility testing compared to broth microdilution (BMD). Genomic resistance determinants were also predicted for each isolate with AMRFinderPlus.

**Results:**

Categorical agreement (CA) of the Sensititre panel compared to BMD was 95.8%, 90.1%, and 95.8% for CZA, CT, and IMR, respectively. CA of the Phoenix panel was 83.0% for CZA and 85.7% for CT. The Phoenix panel was biased toward under-calling CZA resistance while error rates were acceptable for CT by the error-rate-bound method. AMRFinderPlus identified acquired β-lactamases in 4.6% of first-time patient isolates. CA of genotype with BMD was 74.9% for CZA, 91.9% for CT, and 90.7% for IMR. However, all agents had unacceptably high VME rates using genotypic testing because many phenotypically resistant isolates had no identifiable genotypic resistance determinants.

**Conclusions:**

The Sensititre panel met standard acceptance criteria while the Phoenix panel had low CA for all tested BL/BLIs compared to BMD. Acquired β-lactamases were a rare cause of BL/BLI resistance. Further understanding of resistance mechanisms is required before phenotypic BL/BLI resistance can be reliably predicted from genotype, especially in settings where prevalence of acquired β-lactamases is low.

## Teaser

Two commercial susceptibility testing systems were compared to broth microdilution for testing of newer antipseudomonal β-lactam/β-lactamase inhibitor (BL/BLI) combinations against MDR *Pseudomonas aeruginosa* clinical isolates. Prediction of phenotypic BL/BLI susceptibilities from genotype was also assessed using whole-genome sequencing and AMRFinderPlus.

*Pseudomonas aeruginosa* has an exceptional ability to rapidly develop antimicrobial resistance (AMR) through horizontally acquired resistance genes as well as through mutational adaptation and altered expression of chromosomally encoded resistance determinants [1–3]. Infections due to *P. aeruginosa* with multidrug resistance (MDR) phenotypes are associated with increased mortality [4, 5], and carbapenem-resistant *P. aeruginosa* is recognized as a high-priority pathogen by the World Health Organization [6].

Newer antipseudomonal β-lactam/β-lactamase inhibitor (BL/BLI) combinations are recommended for treatment of MDR *P. aeruginosa* infections with limited first-line treatment options by both the Infectious Diseases Society of America and the European Society of Clinical Microbiology and Infectious Diseases [7, 8]. These BL/BLIs include ceftazidime-avibactam (CZA), ceftolozane-tazobactam (CT), and imipenem-relebactam (IMR).

While the newer BL/BLIs have become invaluable tools to treat MDR *P. aeruginosa*, resistance to these agents has been increasingly reported worldwide [1, 2]. Oliver et al recently published comprehensive reviews on the known mechanisms of resistance to CZA, CT, and IMR [1, 2]. Acquired, inhibitor-resistant β-lactamases are important mechanisms of BL/BLI resistance in many parts of the world; however, these acquired β-lactamases remain rare in North American *P. aeruginosa* isolates [9, 10]. In the absence of acquired β-lactamases, BL/BLI resistance appears to be largely driven by adaptive chromosomal mutations [1, 2]. Resistance to CT and CZA has been particularly associated with Ω-loop mutations in the intrinsic *Pseudomonas*-derived cephalosporinase (PDC) [1, 2]. PDC overexpression can result in low-level CT resistance [11]. PBP3 mutations interfere with β-lactam binding and can result in resistance to CZA, CT, and IMR [12]. CZA and IMR resistance have been linked to overexpression of the MexAB-OprM efflux pump [1, 2].

As the clinical demand for novel antipseudomonal BL/BLIs grows, clinical microbiology laboratories will encounter increasing pressure to provide accurate antimicrobial susceptibility testing (AST) results for these agents in a timely manner [13]. Since CZA, CT, and IMR are tier 3 antipseudomonal antibiotics per current CLSI guidance [14], many laboratories perform AST only on MDR isolates and phenotypic testing may not be available in-house. Results of referred-out AST can take several days, by which time an empiric course may already be completed or the patient may have decompensated on an ineffective regimen. We therefore sought to evaluate the performance of in-house CZA, CT, and IMR phenotypic AST on two commercial platforms compared to referred-out broth microdilution (BMD) results with a large panel of consecutive MDR *P. aeruginosa* clinical isolates collected between 2023 and 2024 from Toronto, Canada.

In Canada, a national surveillance program for MDR *P. aeruginosa* is not mandated, and screening for carbapenemase-producing *P. aeruginosa* is not a routine aspect of infection control practices. Consequently, little is known about the epidemiology and mechanisms of resistance to CZA, CT, and IMR in Canada. We therefore also used whole genome sequencing (WGS) and a publicly available AMR database to identify carbapenemases and other known genotypic mechanisms of BL/BLI resistance. We also evaluated the correlation of genotype with phenotypic susceptibilities.

## METHODS

### Bacterial Strains

We cultured 6081 *P. aeruginosa* clinical isolates from blood culture, urine, respiratory, and wound specimens obtained from 3522 patients in 2023 and 2024. Isolates were identified by MALDI-TOF mass spectrometry with the Bruker MALDI Biotyper (MBT Compass Library 2023 revision P). Routine susceptibility testing was performed with the NMIC-451 Gram negative panel on the BD Phoenix M50 or by standard disc diffusion per the laboratory's standard operating procedures. Isolates were considered MDR and included in the study if they were nonsusceptible to ceftazidime, ciprofloxacin, and piperacillin-tazobactam. Overall, a total of 186 MDR *P. aeruginosa* clinical isolates were isolated from 131 patients during the study period, of which 183 isolates from 130 patients had BMD results.

### Reference Broth Microdilution

MDR isolates were referred to the provincial reference laboratory for CZA, CT, and IMR susceptibility testing by reference BMD. BMD was performed once per isolate. MIC calling ranges were ≤4/4 to ≥32/4 µg/mL for CZA and ≤1/4 to ≥16/4 µg/mL for both CT and IMR on the BMD panels.

### Sensititre CAN1MSTF Panel Evaluation

Susceptibility testing for CZA, CT, and IMR was performed in-house with a custom Sensititre CAN1MSTF panel (ThermoFisher Scientific) as per the manufacturer's recommendations. MIC calling ranges were ≤4/4 to >16/4 µg/mL for CZA and ≤1/4 to >8/4 µg/mL for both CT and IMR. Panels were read using the Sensititre Vizion Digital MIC Viewing System (ThermoFisher Scientific), and any uncertain MIC reads were resolved with the mirrored Sensititre manual viewbox (ThermoFisher Scientific). Routine QC was performed per the manufacturer's recommendations.

### Phoenix NMIC-451 Panel Evaluation

Susceptibility testing for CZA and CT was performed on the BD Phoenix M50 automated susceptibility testing instrument with the Phoenix NMIC-451 panel as per the manufacturer's recommendations. MIC calling ranges were ≤0.25/4 to >8/4 µg/mL for CZA and ≤1/4 to >8/4 µg/mL for CT. IMR was not on the Phoenix NMIC-451 panel and could not be assessed. Routine QC was performed per the manufacturer's recommendations.

### Data Analysis and Discrepancy Resolution for Phenotypic Testing

Categorical interpretation of MICs was performed using CLSI M100-Ed35 clinical breakpoints (Supplementary Table 1) [14]. Essential agreement (EA), categorical agreement (CA), and rates of very major errors (VME), major errors (ME), and minor errors (MIN) errors were evaluated for both the Sensititre and the Phoenix panels using BMD as the comparator using standard acceptance criteria per CLSI M52 [15]. Errors were also analyzed using the error-rate-bound method as previously recommended given that >20% of isolates were within ± 1 log_2_ dilution of the BL/BLI breakpoints [16]. For discrepancy resolution, Sensititre or Phoenix testing was repeated in triplicate when one of these systems yielded a VME or a ME. Repeat testing was performed once for isolates with a MIN. Essential errors were not repeated if they did not lead to a categorical error.

### Whole Genome Sequencing

Total nucleic acid extraction was performed on EasyMAG (bioMérieux) followed by WGS on the Illumina MiniSeq (2 × 150 bp paired end) with mean coverage depth of 70.1 (interquartile range 51.5–93.7). Sequence quality control and assembly were performed with Bactopia v3.1 using default parameters [17]. Reads were trimmed for quality and adapter removal, filtered using fastp v0.23.4 [18], and then assembled using shovill v.1.0 (https://github.com/tseemann/shovill). To check for contamination and to confirm the taxonomy of each sequenced isolate, assemblies were analyzed using sourmash v4.8.2 [19] against GTDB release 207 and mash v2.3 [20] against NCBI RefSeq release 88. Assemblies were annotated using prokka v1.14.6 [21]. In silico multilocus sequence typing (MLST) was performed using mlst v2.23.0 (www.github.com/tseemann/mlst) with the *P. aeruginosa* scheme from www.pubmlst.org (downloaded 2025-03-18). A core genome based on loci present in ≥99% of all isolates was estimated using the Bactopia v3.1 pangenome workflow with Panaroo v1.5.0 [22]. ClonalframeML v1.12 [23] was used to mask potentially recombinant sites in the concatenated alignment for core loci, and a maximum likelihood phylogeny was inferred from single nucleotide variants (SNV) in this alignment using IQ-tree v2.2.2.7 with default workflow parameters [24].

### Identification of BL/BLI Resistance Determinants

AMR genes and mutations were predicted using AMRFinderPlus v3.12.8 [25]. Isolates were considered genotypically resistant to a given BL/BLI if the isolate carried a resistance gene or mutation previously reported in the literature to confer resistance to that antibiotic. CA between BMD phenotype and genotype was defined as an isolate testing phenotypically susceptible and having no known resistance determinants or an isolate testing phenotypically resistant and having a known resistance determinant. A VME occurred when the genotype predicted false susceptibility (the BMD result was resistant) while a ME occurred when the genotype predicted false resistance (the BMD result was susceptible). Phenotypically intermediate isolates were excluded from this analysis.

## Supplementary Material

jiaf585_Supplementary_Data

## Figures and Tables

**Table 1.** Summary Reference BMD Susceptibility Data of 183 MDR *P. aeruginosa* Isolates

Antibiotic	Total Isolates	MIC_50_ (µg/mL)	MIC_90_ (µg/mL)	MIC Range (µg/mL)	S		I		R	
					N	%	N	%	N	%
CZA	183	8/4	≥32/4	≤4/4 to ≥32/4	125	68.3	n/a	n/a	58	31.7
CT	183	2/4	8/4	≤1/4 to ≥16/4	147	80.3	23	12.6	13	7.1
IPM^a^	167	8	>8	≤1 to >8	63	37.7	19	11.4	85	50.9
IMR	183	≤1/4	4/4	≤1/4 to ≥16/4	143	78.1	21	11.5	19	10.4

^a^Imipenem (IPM) susceptibilities by BMD are also shown to facilitate comparison with IMR. IPM testing was not performed on 16 isolates.

**Table 2.** Cross-resistance of Novel Antipseudomonal BL/BLI Agents in First-time MDR *P. aeruginosa* Isolates

	CZA S		CT S		IMR S		S To At Least One Agent	
	*N*	%	*N*	%	*N*	%	*N*	%
CZA I/R (*N* = 37)	…	…	22	59.5	22	59.5	26	70.3
CT I/R (*N* = 23)	8	34.8	…	…	10	43.5	12	52.2
IMR I/R (*N* = 27)	12	44.4	14	51.9	…	…	16	59.3

**Table 3.** Overall Performance of the Sensititre CAN1MSTF Panel

Antibiotic	EA (%)	CA (%)	VME (%)	ME (%)	MIN (%)
CZA	72/72 (100.0%)	69/72 (95.8%)	1/20 (5.0%)	2/52 (3.8%)	n/a
CT	68/71 (95.8%)	64/71 (90.1%)	0/2 (0.0%)	0/61 (0.0%)	7/71 (9.9%)
IMR	72/72 (100.0%)	69/72 (95.8%)	0/6 (0.0%)	0/58 (0.0%)	3/72 (4.2%)

**Table 4.** Overall Performance^a^ of the Phoenix NMIC-451 Panel

Antibiotic	EA (%)	CA (%)	VME (%)	ME (%)	MIN (%)
CZA	164/182 (90.1%)	151/182 ***(83.0%)***	26/58 ***(44.8%)***	5/124 ***(4.0%)***	n/a
CT	156/182 ***(85.7%)***	156/182 ***(85.7%)***	2/13 ***(15.4%)***	0/146 (0.0%)	24/182 ***(13.2%)***

^a^Values in bolded italics did not meet standard CLSI acceptance criteria [15].

**Figure 1.**
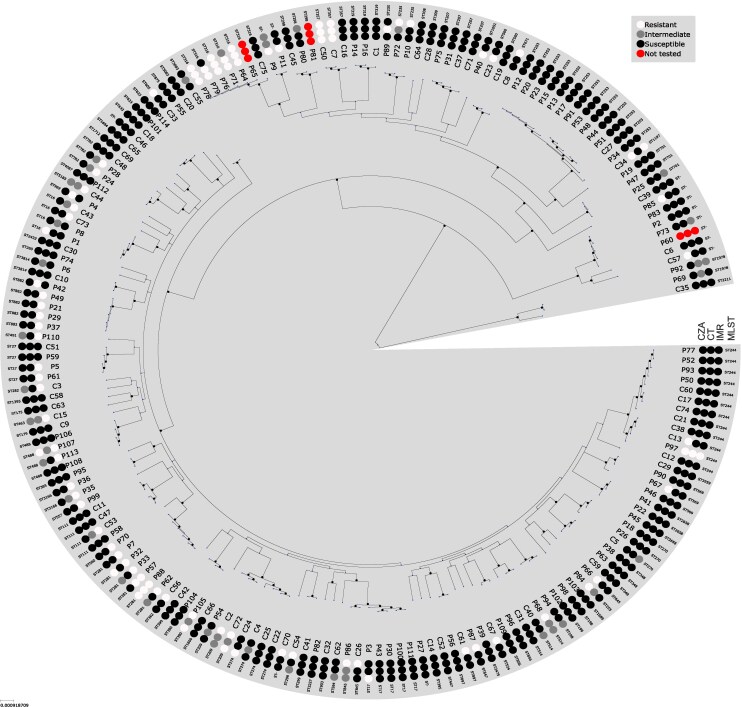
Maximum likelihood core genome SNV phylogeny. The core genome of the MDR *P. aeruginosa* isolates was 5.2 Mbp and consisted of 4355 loci. Isolates are labeled with the isolate number and the in silico predicted sequence type (ST). Susceptibilities by BMD to CZA (inner ring), CT (middle ring), and IMR (outer ring) are represented using colored circles: resistant—white; intermediate—gray; susceptible—black; red—not tested.

**Table 5.** Ability of Genotype to Differentiate Phenotypically Susceptible and Resistant Isolates^a^

Antibiotic	CA (%)	VME (%)	ME (%)
CZA	137/183 ***(74.9%)***	43/58 ***(74.1%)***	3/125 (2.4%)
CT	147/160^b^ (91.9%)	2/13 ***(15.4%)***	11/147 ***(7.5%)***
IMR	147/162^b^ (90.7%)	14/19 ***(73.7%)***	1/143 (0.7%)

^a^Values in bolded italics did not meet standard CLSI acceptance criteria [15].

^b^Isolates that tested intermediate by BMD were excluded because an intermediate category was not defined for genotypic AST.

**Figure 2.**
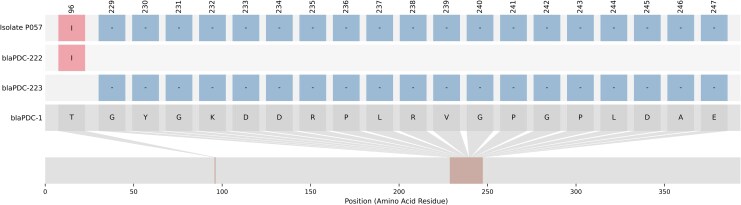
Comparison of amino acid changes in the PDC allele from isolate P057, PDC-222 (RefSeq WP_100931732), and PDC-223 (RefSeq WP_100931733) relative to wild-type PDC-1 (RefSeq NP_252799). The image was generated with snipit v1.6 (https://github.com/aineniamh/snipit) and inkscape v1.4.2 (https://gitlab.com/inkscape/vectors/content/-/issues/117?version=2767059).

## Data Availability

WGS data have been deposited in GenBank under BioProject PRJNA1285505.

## RESULTS

### Phenotypic Characteristics of the MDR *P. aeruginosa* Isolate Collection

Over the course of routine patient care in 2023 (Supplementary Table 2) and 2024 (Supplementary Table 3), we collected 186 clinical isolates of MDR *P. aeruginosa* from 131 unique patients. Overall, 3.7% of patients (131/3522) over the study period had at least one MDR *P. aeruginosa* isolate. CZA, CT, and IMR susceptibilities by BMD were available for 183 MDR *P. aeruginosa* isolates (from 130 patients) (Table 1).

Considering only the first MDR *P. aeruginosa* isolate from a given patient during the study period (*N* = 130), CT was the most active agent with 82.3% (107/130) of MDR *P. aeruginosa* isolates testing susceptible, followed by IMR (79.2%, 103/130) and CZA (71.5%, 93/130) (Supplementary Table 4). Cross-resistance was relatively common amongst the three agents for first-time isolates (Table 2). 8.5% (11/130) of isolates were nonsusceptible to all three novel BL/BLI combinations. CT nonsusceptibility portended a higher likelihood that no BL/BLI agent would be active.

### Evaluation of the Sensititre CAN1MSTF Panel

Using BMD results from the reference laboratory as the comparator, we assessed in-house performance of the custom Sensititre CAN1MSTF panel for CZA, CT, and IMR against the 72 clinical isolates from 2023 (Table 3 for CLSI M52 standard acceptance criteria and Supplementary Table 5 for error-rate-bound method). Scattergrams are presented in Supplementary Table 6.

Initial testing of CZA yielded 6 VME and 6 ME. Repeat testing resolved all but 1 VME and 2 ME. The final performance for CZA was acceptable with 100.0% EA and 95.8% CA.

For CT, isolate C051 had invalid Sensititre results after two attempts and was excluded. We initially had 8 MIN with no VME or ME. Only one MIN resolved after repeat testing, resulting in a MIN rate of 9.9%. Performance for CT was overall acceptable with 95.8% EA and 90.1% CA.

Finally, IMR testing initially resulted in 2 ME, 4 MIN, and no VME. Repeat testing resolved all but 3 MIN, which were all within EA. Final performance for IMR was 100.0% EA and 95.8% CA.

### Evaluation of the Phoenix NMIC-451 Panel

We next assessed performance of the Phoenix NMIC-451 panel for CZA and CT against all 183 clinical isolates with paired BMD results (Table 4 for CLSI M52 standard acceptance criteria and Supplementary Table 5 for error-rate-bound method). Scattergrams are presented in Supplementary Table 6. Of note, there were no major differences in Phoenix performance with the 2023 isolate subgroup compared to the 2024 subgroup (data not shown). Isolate C038 (CZA and CT susceptible by BMD) was excluded for repeatedly terminated runs on the Phoenix system.

Initial CZA testing led to 28 VME and 10 ME, with resolution of only 2 VME and 5 ME upon repeated testing. Most VME (16/26; 61.5%) and all ME were within EA. Although EA was acceptable at 90.1%, the VME rate was unacceptably high even when assessed with the error-rate-bound method (Supplementary Table 5).

CT susceptibility testing initially resulted in 3 VME, 2 ME, and 24 MIN. Upon repeat Phoenix testing of the discrepant isolates, there remained 2 VME, no ME, and 15 MIN. Although EA and CA were both <90%, the VME and MIN rates were acceptable by the error-rate-bound method (Supplementary Table 5).

### Genomic Characteristics of the MDR *P. aeruginosa* Isolate Collection

WGS was performed on all 186 isolates, and phylogeny is presented in Figure 1. Several isolates (27/186; 14.5%) belonged to globally important, clonal epidemic lineages (ST111, ST175, ST235, ST244, ST298, ST308, and ST357) that are at high-risk for development of multidrug resistance [1, 26]. To our knowledge, this represents the first evidence for expansion of ST308 and ST357 into North America.

### Identification of Resistance Determinants With AMRFinderPlus

We used the NCBI AMRFinderPlus tool to characterize the resistomes (Supplementary Table 7) of all 183 isolates that underwent BMD testing. Since we were specifically interested in mechanisms of resistance to CZA, CT, and IMR, we then filtered the AMRFinderPlus output to include only those determinants that are involved in β-lactam resistance (Supplementary Table 8). In patients with multiple isolates, we did not observe any notable differences in AMRFinderPlus output between isolates that would explain differences in susceptibility patterns.

### Acquired β-lactamases

Only 3 isolates harbored carbapenemases, which is consistent with prior findings that acquired carbapenemases, are uncommon in MDR *P. aeruginosa* isolates from North America [9, 10]. Nonsusceptibility of isolate P072 to CT was the result of KPC-2, a class A carbapenemase that is inhibited by avibactam and relebactam [1, 27]. Two isolates had metallo-β-lactamases (NDM-1 in C007 and VIM-5 in C050), which accounted for the high-level resistance that we observed to all tested BL/BLIs in these two isolates [1]. C007 also carried the class A ESBL enzyme VEB-9 while C050 also harbored VEB-14 and a class D OXA-10-like enzyme [28]. Interestingly, both C007 and C050 belonged to the high-risk ST357 lineage.

Isolates P064, P065, P071, P076, P078, and P079 (all from the same patient) carried a rare class C enzyme called PAC-1 [29]. This enzyme is known to confer high-level resistance to CZA and CT but not to carbapenems. We also identified the class A ESBL enzyme, GES-1, in isolate P089, which was CT resistant but CZA and IMR susceptible. Consistent with the isolates phenotype, GES-1 can hydrolyze ceftolozane but not carbapenems, and it is inhibited by avibactam [30, 31]. Of note, a poorly characterized RSC-1-like enzyme was incidentally identified in isolate P101—this class C β-lactamase is not known to confer resistance to the newer BL/BLIs, and all these agents tested susceptible in this isolate. Overall, acquired β-lactamases were rare and accounted for phenotypic resistance in 12.1% (7/58), 61.5% (8/13), and 10.5% (2/19) of CZA, CT, and IMR resistant isolates, respectively.

### Intrinsic PDC Variants

PDC alleles with Ω-loop mutations are particularly important for clinical resistance to CZA and CT in the absence of horizontally acquired β-lactamases [1, 2]. Surprisingly, we did not identify any PDC variants in our isolates that are known to confer resistance to CZA or CT [1, 32–38].

### PDC Derepression

PDC hyperproduction, either via mutations in the *ampR* transcriptional regulator or in components of the cell-wall recycling pathway (eg, *dacB*, *ampD*, etc), can lead to CT resistance [11]. Four isolates (C020, C026, P086, and P104) carried a D135G mutation in *ampR* that was identified by AMRFinderPlus. Mutation of D135 is known to lead to increased CT MICs through overexpression of PDC [11]; however, this mutation was not fully penetrant in our isolates since all isolates with the *ampR*_D135G_ mutation tested CT susceptible except for P086. AMRFinderPlus also identified the *dacB*_T428P_ mutation in three isolates (P048, P053, and P091) and *ampD* mutations in two isolates (C056 and P068). Again, these mutations did not predict clinical resistance to CT since all these isolates were CT susceptible except isolate P068.

### PBP3 Mutations

PBP3 (encoded by *ftsI*) is an essential penicillin-binding protein in *P. aeruginosa*, and *ftsI* mutations that decrease β-lactam binding affinity have been associated with resistance to CZA, CT and IMR [12, 39, 40]. AMRFinderPlus did identify *ftsI*_R504C_ mutations in seven isolates (C054, P032, P033, P057, P062, P088, and P097). While all 7 of these isolates tested CZA resistant and 6 tested IMR nonsusceptible, and only 3 were CT nonsusceptible.

### MexAB-OprM Overexpression

Overexpression of the constitutive MexAB-OprM efflux pump has been associated with CZA resistance [41, 42]. Loss of MexR repressor function is a classic mechanism of MexAB-OprM derepression. AMRFinderPlus identified a *mexR* nonsense mutation (Q49*) in four isolates (P002, P073, P083, and P085), of which only isolate P085 was CZA resistant. While IPM is not a substrate of the MexAB-OprM efflux pump, overexpression of MexAB-OprM in an OprD deficient background can give rise to IMR resistance [43, 44]. No concomitant *oprD* mutation was identified in these isolates by AMRFinderPlus, and none of the isolates with *mexR*_Q49*_ were resistant to IMR.

### Prediction of BL/BLI AST Phenotype With Genotypic Determinants Identified by AMRFinderPlus

The correlation of genotype with phenotypic AST by BMD is summarized in Table 5. Scattergrams are presented in Supplementary Table 9. CA for CT and IMR were both >90%. CA for CZA was lower at 74.9%. The VME rate was unacceptably high for all tested agents, suggesting that the full spectrum of genetic mechanisms that likely underlie resistance to CZA, CT, and IMR in our isolate collections are not well characterized or fully represented in reference databases.

### Identification of a Novel Hybrid PDC Variant Conferring CZA and CT Resistance in Isolate P057

Isolate P057 was resistant to CZA and CT, but susceptible to IPM and IMR. Interestingly, the PDC in this isolate did not match any known PDC variants in the AMRFinderDatabase, so we sought to characterize the PDC allele of this isolate further. NCBI BLASTp analysis of the PDC sequence from isolate P057 had no perfect hits, but the closest matches were to PDC-222 (95.2% identity) and PDC-223 (99.7% identity). PDC-222 (PDC-1_T96I_) and PDC-223 (PDC-1_ΔG229-E247_) are known expanded-spectrum AmpC enzymes with activity against CZA and CT but not carbapenems [33, 34, 35, 45]. The T96I and ΔG229-E247 mutations confer reduced susceptibility to avibactam inhibition, and they also open the Ω-loop entrance to accommodate ceftolozane and other β-lactams with bulkier side chains [33]. Interestingly, the PDC allele from isolate P057 carried both the T96I and ΔG229-E247 mutations (Figure 2). To our knowledge, this combination of mutations in the same PDC variant has not been previously reported.

## DISCUSSION

CZA, CT, and IMR are increasingly required for treatment of *P. aeruginosa* with multidrug or difficult-to-treat resistant phenotypes [7, 8]. Unfortunately, but perhaps unsurprisingly, clinicians now face the challenge of treating MDR *P. aeruginosa* isolates that have also developed resistance to these antipseudomonal BL/BLIs [1, 2]. Timely access to accurate susceptibility testing is therefore important when managing patients with MDR *P. aeruginosa* infections.

In this work, we provided the first independent clinical evaluation of the Sensititre CAN1MSTF and Phoenix NMIC-451 panels for susceptibility testing of the novel antipseudomonal BL/BLIs compared to BMD against MDR *P. aeruginosa* clinical isolates. The Sensititre platform had acceptable performance characteristics for CZA, CT, and IMR susceptibility testing compared to reference BMD. The Phoenix system was acceptable for CT testing when evaluated with the error-rate-bound method. However, the VME rate of CZA was unacceptably high with the Phoenix panel despite an EA ≥90%. The lack of an intermediate category to buffer expected fluctuations in MIC testing has made CZA testing challenging for clinical laboratories [13]. A major limitation of our work was that BMD was performed once off-site by the provincial reference laboratory. As a result, the same inoculum could not be used for testing by BMD and the commercial systems. Moreover, we were unable to repeat BMD to assist with discrepancy resolution, so we cannot exclude the possibility that errors arose due to BMD inaccuracies. It is important to note, however, that most clinical laboratories are unable to perform BMD, so this limitation represents the reality of most laboratories seeking to verify commercial antibiotic susceptibility testing systems. Although our laboratory serves multiple health networks with a highly diverse patient population, our evaluation was limited by a single geographic catchment for the isolates. Future work will seek to evaluate the multicenter performance of these and other commercial test systems for novel antipseudomonal BL/BLI testing with contemporary Canadian isolates.

Very little is known about the epidemiology of *P. aeruginosa* resistance to novel BL/BLIs in Canada. In our collection of MDR *P. aeruginosa*, the susceptibility of first-time isolates was highest for CT followed by IMR and then by CZA. This pattern is similar to that described in the United States, where rates of carbapenemase-producing *P. aeruginosa* remain relatively low [7, 10]. Almost a tenth of first-time isolates were nonsusceptible to all three BL/BLIs, which further reinforces the necessity of timely, in-house susceptibility testing in our setting. Of note, we identified isolates that belonged to several globally important clones that are at high-risk of developing resistance—in particular, we reported the expansion of ST308 and ST357 into North America for the first time.

We used WGS to identify resistance genes and mutations that could explain the burden of BL/BLI resistance in our isolate collection. Consistent with prior data indicating that carbapenemase-producing *P. aeruginosa* are rare in Canada, acquired β-lactamases were not a common source of BL/BLI resistance in our isolate collection [9]. However, more than half of the CT resistant isolates did carry an acquired β-lactamase, so laboratories could consider targeted screening of *P. aeruginosa* isolates that are resistant to CT for epidemiological and infection control purposes as previously suggested by others [46]. Unfortunately, many of the phenotypically nonsusceptible isolates had no genotypic explanation identified with AMRFinderPlus. These observations raise two important considerations. First, BL/BLI resistance is a complex and multigenic phenotype that is incompletely understood and not fully curated in AMR reference databases. Further understanding of BL/BLI resistance mechanisms is required in settings where the prevalence of acquired β-lactamases is low. Secondly, our findings echo prior observations that genotype imperfectly predicts *P. aeruginosa* phenotypic resistance to many antimicrobials [47–49]. Accurate culture-independent prediction of antipseudomonal BL/BLI resistance may require a multiomics approach that considers the genome, transcriptome, proteome, and metabolome of this highly versatile and adaptive pathogen.

## Supplementary Data

Supplementary materials are available at *The Journal of Infectious Diseases* online (http://jid.oxfordjournals.org/). Supplementary materials consist of data provided by the author that are published to benefit the reader. The posted materials are not copyedited. The contents of all supplementary data are the sole responsibility of the authors. Questions or messages regarding errors should be addressed to the author.
